# Traumatic Simultaneous Bilateral Knee Dislocation: A Case Report

**DOI:** 10.7759/cureus.18989

**Published:** 2021-10-23

**Authors:** Benjamin T Harris, Elizabeth A Eichman, Matthew T Burrus

**Affiliations:** 1 College of Osteopathic Medicine, University of New England, Biddeford, USA; 2 School of Medicine, Texas Tech University Health Sciences Center, Lubbock, USA; 3 Orthopedic Surgery, Orthopedic Associates of Central Texas, Austin, USA

**Keywords:** ortho surgery, ligament reconstruction, knee dislocation, popliteal artery, trauma

## Abstract

Bilateral knee dislocation is an extremely rare and devastating injury to the knee joint. This injury is a true emergency as concomitant injuries may threaten life and limb. Here, we report the case of a 26-year-old male patient who sustained bilateral knee dislocation due to a high-velocity motor vehicle accident. In this case, the patient suffered a feared complication associated with knee dislocation, popliteal artery disruption with peroneal nerve damage. Abdominal and skeletal injuries were also discovered and treated appropriately by a multidisciplinary team. A staged surgical approach to ligament reconstruction was used without any postoperative complication. Postoperatively, the patient successfully returned to his preoperative activity level.

## Introduction

Knee dislocation is a rare and devastating injury with an estimated prevalence of 0.02-0.2% of reported orthopedic injuries. A dislocation occurs bilaterally in only 5% of all knee dislocations. The common causes of knee dislocation are high-velocity trauma such as a motor vehicle accident (50%), low-velocity sports injuries (33%), and ultralow-velocity injuries (12%) such as a fall. The most common injury pattern is the anterior dislocation of the tibia with respect to the femur (40%), and the second most common injury pattern is a posterior dislocation (30%) [1].

Knee dislocation involves complete disruption of the integrity of the tibiofemoral articulation and is commonly reported in terms of the Schenck classification scheme. The Schenck classification was later modified by Yu to include arterial and neural injury designation, C and N, respectively. KD-I is associated with a single cruciate injury. KD-II is a bicruciate injury. KD-III represents a bicruciate injury with either posteromedial or posterolateral disruption. KD-IV is a bicruciate injury with both posteromedial and posterolateral disruption. KD-V signifies a knee dislocation with an associated fracture. KD-III is the most common injury in terms of the Schenck classification scheme [1].

Knee dislocation may result in injuries in addition to ligamentous damage such as popliteal artery disruption, peroneal nerve damage, knee joint fractures, meniscal pathology, patellar ligament tears, and iliotibial tract injury [2]. Patients who sustain bilateral knee dislocations have demonstrated increased morbidity when compared to unilateral knee dislocation. Bilateral knee dislocation patients have significantly higher Injury Severity Score and New Injury Severity Score values compared to a unilateral dislocation which underlies the seriousness of a bilateral injury [3]. Knee dislocation patients are at risk of life-threatening concomitant injuries. One study documented that 27% of knee dislocation patients presented with life-threatening injuries to the head (subdural hematoma), chest (pneumothorax, flail chest), or abdomen (ruptured viscera) [4]. Thus, the standard Advanced Trauma and Life Support protocol should be followed in patients who suffer a knee dislocation as early intervention may be life-saving.

In the majority of situations, ligament reconstruction is recommended due to the predictable poor function with nonoperative management. A systematic review involving 916 patients with knee multiligament injuries, such as a knee dislocation, concluded that operative management leads to favorable outcomes in terms of functional outcome, instability, contracture, and return to activity [5]. Preoperatively, it is imperative to counsel a knee dislocation patient about the high complication rate and possible long-term consequences of their injury as in any other serious injury. Arthrofibrosis is a common complication and requires surgical treatment in 29% of patients. Posttraumatic osteoarthritis is reported in 29.6-53% of knees possibly due to acute cartilage damage or residual laxity [6].

## Case presentation

A healthy 26-year-old male with no pertinent medical, surgical, or family history was ejected from a vehicle traveling at highway speeds. At the hospital, he arrived with a Glasgow Coma Score of 3, was intubated, and diagnosed with bilateral knee dislocation, left popliteal artery disruption, left peroneal nerve palsy, multiple pelvic fractures, right acetabular and pubic rami fractures, intraperitoneal bladder rupture, grade II splenic injury, multiple rib fractures, right-sided pneumothorax, and left scapular body fracture.

General surgery initially evaluated the patient and performed a splenectomy and bladder repair. A vascular surgeon then bypassed the left popliteal artery disruption utilizing the contralateral saphenous vein as a graft. The on-call orthopedic surgeon stabilized the left knee with external fixation and treated a developing compartment syndrome with a four-compartment fasciotomy. Two days later, a second orthopedic surgery consisted of right trans-sacral screw placement, open reduction and fixation of the acetabular fractures, and fasciotomy wound closure with vacuum dressing. This wound was later skin grafted by a plastic surgeon for an optimal outcome.

**Figure 1:**
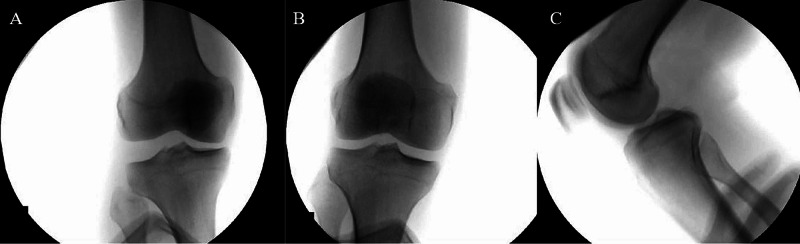
Preoperative right knee stress radiographs. Right knee preoperative stress radiographs demonstrate LCL laxity (A), an intact MCL (B), and excess tibial posterior translation (C). MRI was not available preoperatively due to the left knee external fixation. LCL: lateral collateral ligament; MCL: medial collateral ligament; MRI: magnetic resonance imaging

The attending orthopedic surgeon evaluated the left knee three days after injury and noted a knee-spanning external fixator in place with normal distal pulses. Neurologically, the sensation was overall decreased with the sensation only to deep pressure at the dorsum and lateral aspect of his foot. The right knee had no neurovascular deficits, a proximal lateral thigh mass, and positive posterior drawer and 3B Lachman examinations. There was greater than 5 mm of opening to varus stress in full extension and 30 degrees of flexion. External fixation precluded MRI scans prior to the first surgical procedure, thus stress radiographs of the right knee were taken and demonstrated significant lateral gapping in addition to the excess posterior tibial translation. The stress radiographs can be seen in Figure 1.

Nonoperative management was discussed with the patient; however, it was not recommended due to the patient’s age and desired activity level. Staged reconstruction was determined to be the optimal treatment option based on a literature review. Staged reconstruction involves acute surgery (within two weeks) of the extra-articular injuries, followed by reconstruction of the injured cruciate(s) once acceptable knee motion has been achieved. The plan was to operate on the right knee first as the left knee required additional time for the popliteal bypass graft to mature. Before proceeding, it was necessary to ensure that previous traumas (bladder, spleen, popliteal artery bypass) were stable and improving. Throughout the patient’s recovery, appropriate communication was maintained with all involved providers.

**Figure 2:**
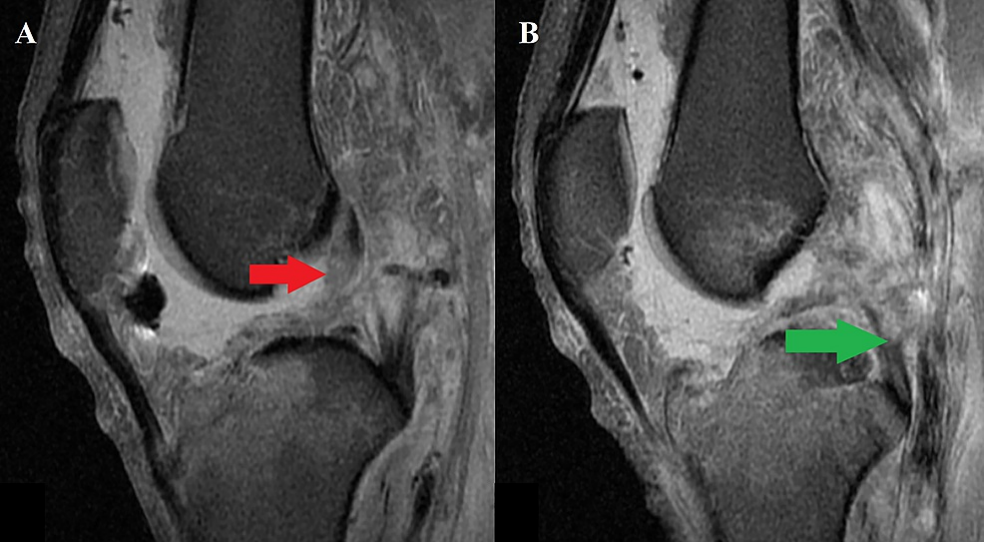
Preoperative MRI of the left knee. Left knee preoperative MRI shows ACL (red arrow) and PCL (green arrow) disruption, large effusion, and significant soft tissue edema. ACL: anterior cruciate ligament; PCL: posterior cruciate ligament; MRI: magnetic resonance imaging

The first surgery included right knee diagnostic arthroscopy, removal of the anterior cruciate ligament (ACL) and posterior cruciate ligament (PCL) remnants, and repair of the medial and lateral meniscus. The lateral collateral ligament (LCL) and popliteus were also reconstructed and reattached, respectively. As suspected clinically, based on a mass in his proximal lateral thigh, the biceps femoris had significantly retracted up into his thigh and was left there as reattachment was not possible. Six weeks following the initial surgery on the right knee, a second procedure included operating on both knees. In the right knee, the ACL (quadriceps tendon autograft) and PCL (allograft) were reconstructed utilizing all-inside techniques. Additionally, a posterolateral corner repair of the LCL, popliteus, and popliteofibular ligament was performed and augmented with allograft. The left knee underwent removal of the external fixation, diagnostic arthroscopy, manipulation, and lysis of adhesions. Intraoperatively, the range of motion (ROM) was 0 to 120 degrees. Four months later, the right knee was doing well, and the patient demonstrated a passive ROM from 0 to 130 degrees, 5/5 knee flexor and extensor strength, no tenderness or effusion, negative Lachman examination, and the ability to stand for a short period of time without his knee braces. However, the patient reported continued left knee instability and reconstruction was recommended. The preoperative MRI on the left knee can be seen in Figure 2. All-inside techniques were used for the left knee ACL (autograft) and PCL (autograft) reconstructions. The patient’s popliteus was intact, leading to LCL reconstruction utilizing an autograft, and not a full posterolateral corner reconstruction. No meniscal or full-thickness chondral pathology was observed during the operation.

**Figure 3:**
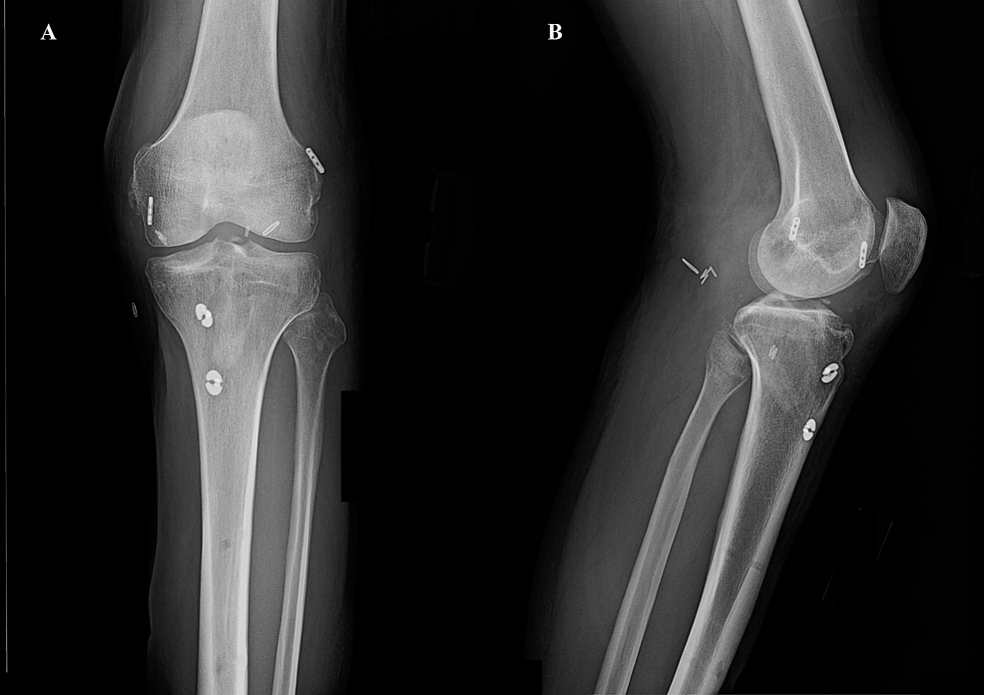
Left knee postoperative radiographs. Final left knee postoperative radiographs demonstrating proper graph fixation placement in the anterior-posterior (A) and lateral (B) views. The vascular clips from the popliteal artery repair can be seen in the lateral image (B).

**Figure 4:**
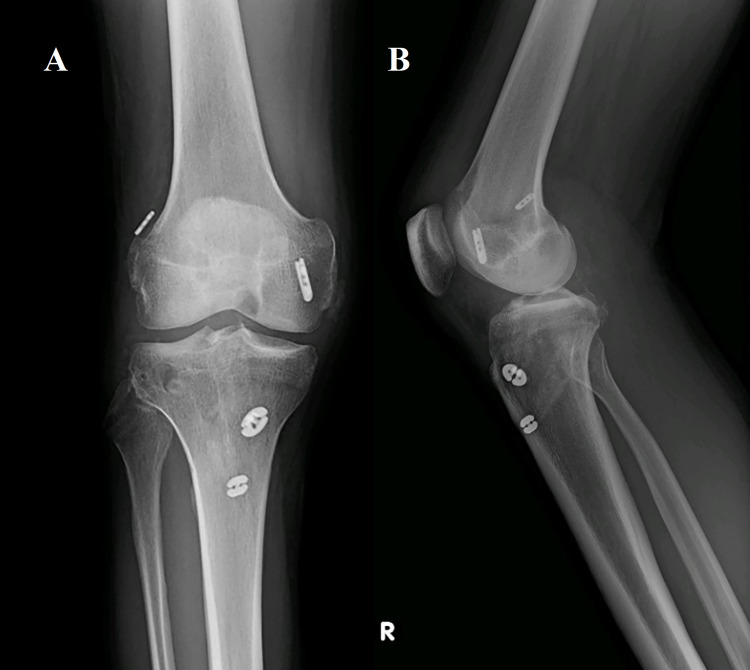
Right knee postoperative radiographs. Final right knee postoperative radiographs in the anterior-posterior (A) and lateral views (B).

Postoperatively, the patient was carefully managed with a strict rehabilitation protocol. After the initial operation, the right knee was limited to 0-90 degrees of ROM by a hinged ACL knee brace. Weight-bearing was limited to toe-touch secondary to the acetabular and pelvic fractures, with progression to weight-bearing as tolerated after three months. After the second procedure, the right knee was limited to 0-90 degrees of ROM in the prone position only with toe-touch weight-bearing for six weeks, and then weight-bearing and ROM were progressed as tolerated. The left knee had no ROM restriction with 50% weight-bearing for six weeks and then was progressed to weight-bearing as tolerated. After the third procedure, the left knee was limited to 0-90 degrees of ROM in the prone position only and no weight-bearing for six weeks with the progression of ROM and weight-bearing as tolerated after that. Postoperative radiographs can be seen in Figure 3 and Figure 4 for the left and right knees, respectively. The patient successfully completed the physical therapy recovery protocol and eventually returned to his desired sport, golf. There was complete resolution of all neurovascular deficits and no evidence of graft disruption. There are no long-term complications to report at three years postoperatively.

## Discussion

Bilateral knee dislocation is a serious injury and a thorough examination may potentially be limb-saving. Quick identification of a vascular injury is essential as ischemia time of more than eight hours prior to vascular repair results in an amputation rate of 86%. The popliteal artery is particularly susceptible to injury during knee dislocation. A recent database analysis of 8,050 knee dislocations estimated the overall risk of vascular injury to be 3.3% (95% confidence interval = 2.9-3.7%), 13% of which required vascular repair. The authors of this study noted that an increase in reported low-velocity knee dislocations, which are less likely to have a vascular injury, may be responsible for the low rate of vascular injury reported in their study compared to the previous literature [7]. A second study of 862 patients with knee dislocations reported 18% sustained vascular injury, and 80% of those required surgical repair [8]. The study demonstrated a wide range in the reported rates of vascular injury and in vascular injuries requiring surgical repair after knee dislocation. Clinically, it is important that providers be aware that male patients aged 20-39 years are the most likely to suffer a vascular injury after knee dislocation and to err on the side of caution in ruling out arterial injury [7].

Management of the ligamentous injury is critical to producing an optimal long-term outcome. Surgical treatment of a knee multiligament injury is advantageous when compared to nonoperative treatment [5]; however, there is no universally accepted recommendation involving the timing, technique, and graft type. Regarding ligament reconstruction techniques, there are numerous options concerning tunnel locations, the number of cruciate tunnels (double versus single), fixation devices, order of fixation, and grafts (allograft versus autograft). The current literature does not provide a clear recommendation, and thus the decisions are made mostly on surgeon preference.

This is a difficult population to study due to the rarity of these injuries and the large variability in injury patterns. However, staged, acute, and delayed reconstruction approaches have been examined. Staged reconstruction is a step-wise process involving acute surgical treatment of the extra-articular injuries followed by cruciate reconstruction once full knee motion has been restored. Supporters of acute reconstruction argue that reconstruction of the cruciate ligaments is vital for the restoration of collateral ligaments and corner structures. Delayed reconstruction allows the knee joint to heal before considering operative treatment and may avoid unnecessary repair of extra-articular structures [6]. The literature demonstrates superior outcomes associated with a staged approach to reconstruction compared to the acute or delayed approaches [9,10]. A systematic review of 396 knees who underwent either staged, acute, or delayed reconstruction found that 79% of the staged cohort achieved either an excellent or good subjective outcome score. The acute and chronic approaches produced an excellent or good outcome 52% and 37% of the time, respectively [9]. A second systematic review presented similar results in terms of excellent or good outcomes among staged (79%), acute (58%), and delayed (46%) reconstruction [10].

At 14 and 18 months postoperatively for the left and right knee, respectively, in our patient, a Knee Injury and Osteoarthritis Outcome Score (KOOS) was completed. The average KOOS values from a study of one-year postoperative knees recovering from multiligament reconstruction are shown for comparison [11] in Table 1.

**Table 1:** Left and right knee results for the patient were recorded at 14 and 18 months, respectively. For comparison, KOOS scores from knees 12 months after multiligament reconstruction are reported. KOOS: Knee Injury and Osteoarthritis Outcome Score. S/p: status post; MLIR: multiligament reconstruction

	KOOS Pain	KOOS Symptoms	KOOS ADL	KOOS Sports	KOOS QOL
Left knee	89	82	84	40	56
Right knee	81	79	82	45	56
Average KOOS 12 months s/p MLIR	74	69	78	36	46

## Conclusions

This case highlights the importance of a thorough initial examination of patients presenting with knee dislocation. If the popliteal artery disruption was initially missed, it is possible that the young patient could have lost his lower leg. Clinically, managing knee dislocation patients requires careful use of appropriate vascular studies with serial examinations as indicated. Mandatory vascular evaluations with arteriograms should be considered in these patients with high-energy mechanisms. Once stabilized, the approach to ligament reconstruction is largely based on surgeon preference. A staged approach to reconstruction was used in this case. The approach included acute surgery (within two weeks of initial injury) of the extra-articular injuries, followed by reconstruction of the injured cruciate(s) once acceptable knee motion had been achieved. There were no long-term complications and the patient successfully returned to his desired sport.
